# Suppression of HSV-1 infection and viral reactivation by CRISPR-Cas9 gene editing in 2D and 3D culture models

**DOI:** 10.1016/j.omtn.2024.102282

**Published:** 2024-07-19

**Authors:** Anna Bellizzi, Senem Çakır, Martina Donadoni, Rahsan Sariyer, Shuren Liao, Hong Liu, Guo-Xiang Ruan, Jennifer Gordon, Kamel Khalili, Ilker K. Sariyer

**Affiliations:** 1 Center for Neurovirology and Gene Editing, Department of Microbiology, Immunology and Inflammation, Temple University Lewis Katz School of Medicine, Philadelphia, PA 19140, USA; 2 Excision BioTherapeutics Inc., 134 Coolidge Avenue, Watertown, MA 02472, USA

**Keywords:** MT: RNA/DNA Editing, cerebral organoids, HSV-1, CRISPR-Cas9, gene editing, latency, reactivation

## Abstract

Although our understanding of herpes simplex virus type 1 (HSV-1) biology has been considerably enhanced, developing therapeutic strategies to eliminate HSV-1 in latently infected individuals remains a public health concern. Current antiviral drugs used for the treatment of HSV-1 complications are not specific and do not address latent infection. We recently developed a CRISPR-Cas9-based gene editing platform to specifically target the HSV-1 genome. In this study, we further used 2D Vero cell culture and 3D human induced pluripotent stem cell-derived cerebral organoid (CO) models to assess the effectiveness of our editing constructs targeting viral ICP0 or ICP27 genes. We found that targeting the ICP0 or ICP27 genes with AAV2-CRISPR-Cas9 vectors in Vero cells drastically suppressed HSV-1 replication. In addition, we productively infected COs with HSV-1, characterized the viral replication kinetics, and established a viral latency model. Finally, we discovered that ICP0- or ICP27-targeting AAV2-CRISPR-Cas9 vector significantly reduced viral rebound in the COs that were latently infected with HSV-1. In summary, our results suggest that CRISPR-Cas9 gene editing of HSV-1 is an efficient therapeutic approach to eliminate the latent viral reservoir and treat HSV-1-associated complications.

## Graphical abstract

**Figure:**
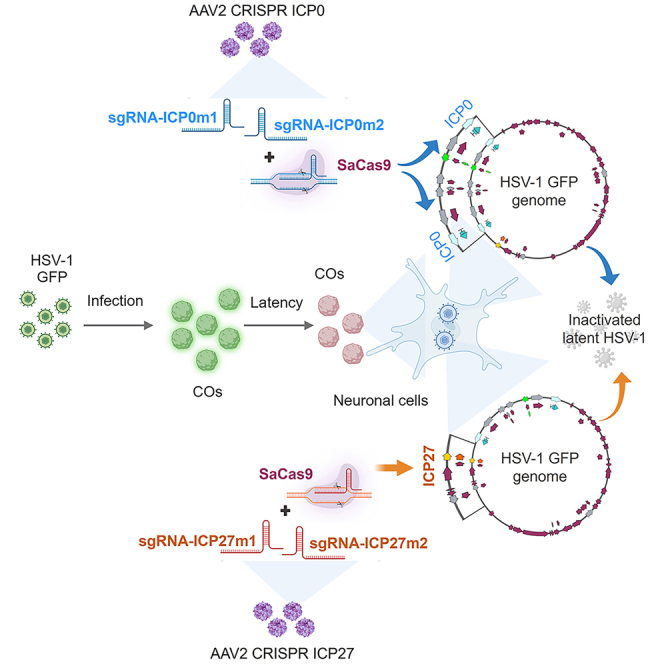

## Teaser

Bellizzi and colleagues have successfully established HSV-1 latency in a 3D culture model of cerebral organoids to prove the efficacy of a gene editing platform based on CRISPR and *Staphylococcus aureus* Cas9 (CRISPR-SaCas9) to specifically target two HSV-1 genes, ICP0 and ICP27, with the suppression of HSV-1 infection.

## Introduction

Herpes simplex virus type 1 (HSV-1) is a member of the *Alpha Herpesviridae* subfamily and consists of a double-stranded DNA genome of ∼152 kbp.^1^ According to the World Health Organization, an estimated 3.7 billion people under the age of 50 years (67%) carry HSV-1.^2^ HSV-1 infection establishes a lifelong latent state within the peripheral nerve ganglia and sensory neurons. HSV-1 can lead to various conditions such as herpes stromal keratitis and meningoencephalitis, which can result in blindness and permanent neurological damage and death, respectively.^1^

The immediate-early genes ICP0 and ICP27 encode proteins that regulate the expression of various early and late viral genes. ICP27 is a multifunctional protein that inhibits cellular pre-mRNA splicing,^3^ stimulates viral early and late gene transcription by recruiting cellular RNA polymerase II to viral genome,^4^^,^^5^ binds and exports viral RNA to the cytoplasm, and stimulates translation of some HSV-1 transcripts by binding translation initiation factors.^6^^,^^7^^,^^8^ On the other hand, ICP0 plays a crucial role in determining whether the HSV virus enters the lytic phase or the latent phase.^9^^,^^10^^,^^11^ Biochemically, ICP0 is an E3 ubiquitin ligase that stimulates the proteasome-dependent degradation of several cellular proteins, inhibiting the cell-mediated restriction of viral gene expression.^12^

Despite several studies on the molecular biology of HSV-1, an *in vitro* HSV-1 infection model of the nervous system is required. This model will help clarify the molecular mechanisms related to viral latency and reactivation at the neuronal level. Recently, 3D neuronal organoid models derived from human induced pluripotent stem cells (hiPSCs) were established to study HSV-1 latency, reactivation, and lytic infection.^13^ Current treatments for HSV-1 infection include the use of antiviral drugs, including acyclovir (ACV). ACV is also widely used to induce viral latency in *in vitro* models. ACV is a nucleoside analog and is commonly used in the clinic as a primary treatment of HSV-1-associated complications.^14^ However, ACV is not able to eradicate the HSV-1 virus from the HSV-1 latency reservoirs. In recent years, the clustered regularly interspaced short palindromic repeats (CRISPR)-Cas9 genome editing strategy has been employed in many studies to target various HSV-1 genes, such as ICP0, ICP4, ICP27, UL8, UL19, UL23, UL27, UL29, UL30, UL35, VP16 (UL48),UL52, US2, and US6 (gD).^15^^,^^16^^,^^17^^,^^18^^,^^19^^,^^20^^,^^21^ We previously used CRISPR-Cas9 to target the HSV-1 genome, which led to excision of the viral genomic DNA (gDNA) and substantial reduction of the viral burden.^22^ In this study, we used the hiPSC-derived cerebral organoids (COs) as a 3D culture model to assess the effectiveness of our CRISPR-SaCas9 gene editing strategy, which specifically targets the HSV-1 ICP0 or ICP27 genes. AAV2 vectors carrying the ICP0- or ICP27-targeting gene editing components led to great suppression of HSV-1 infection in Vero cells and COs. In addition, we characterized the viral replication kinetics in the 3D CO model and established an *in vitro* model of HSV-1 viral latency. Finally, we tested the effect of a CRISPR-Cas9-based HSV-1 editing platform on the reversal of HSV-1 latency in the 3D CO model and found that the anti-HSV-1 gene editing approach significantly reduced the rate of viral rebound from the latent stage. In conclusion, these results suggest that CRISPR-SaCas9-mediated editing of HSV-1 viral genome is an efficient therapeutic strategy to eradicate the latent HSV-1 virus.

## Results

### Suppression of HSV-1 infection in Vero cells transduced with AAV2 vectors expressing SaCas9 and gRNAs targeting ICP0 or ICP27

**Figure 1:**
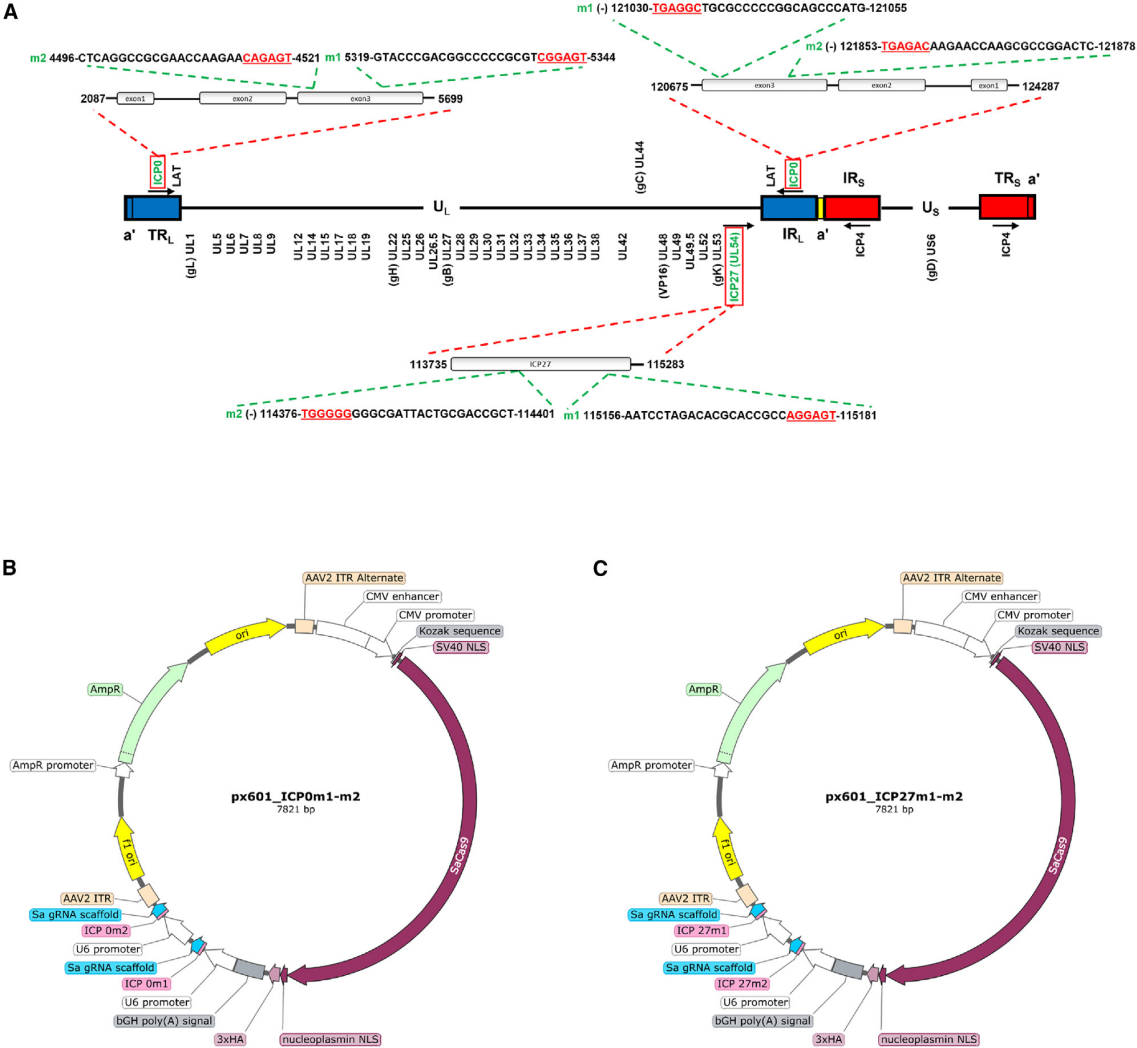
CRISPR-SaCas9 gRNAs specific for ICP0 and ICP27 genes (A) Schematic representation of the HSV-1 genome where positions and nucleotide sequences of gRNAs including PAM (in red and underlined) are shown. The nucleotide positions are referred to RefSeq NC_001806.2 (https://www.ncbi.nlm.nih.gov/nuccore/NC_001806.2). The HSV-1 genome is divided into two major unique fragments (unique long or U_L_ and unique short or U_S_), each flanked by inverted repeats (R_L_ and R_S_, with prefixes I or T denoting internal or terminal) and shorter repeated segments known as the *aʹ* sequence. A graphic representation of the two plasmids packaged in AAV2 particles is shown with (B) pX601_ICP0m1-m2 containing the two gRNAs targeting ICP0 (m1 and m2) and (C) pX601_ICP27m1-m2 containing the two gRNAs targeting ICP27 (m1 and m2). Each of the gRNAs were cloned downstream of a U6 promoter, and each plasmid contains one copy of the SaCas9 gene.

Four gRNAs (two targeting the HSV-1 ICP0 gene and the other two targeting the HSV-1 ICP27 gene) were designed using the guide RNA tool on Benchling for the CRISPR-SaCas9 system, which uses “NNGRRT” as the protospacer adjacent motif (PAM) sequence. The activity scores of gRNAs were calculated according to Doench et al.,^23^ whereas the specificity scores were calculated according to the analysis reported by Hsu et al.^24^ The gRNAs were selected by balancing on-target and off-target scores, with a higher priority giving to specificity in order to minimize potential off-target activity on the human DNA genome. The positions of these gRNAs are shown in Figure 1A, where the PAM sequences are highlighted in red and underlined. The sequences of the four selected gRNAs are also listed in Table S1. The selected gRNAs were then cloned into the pX601 vector expressing SaCas9 under a CMV promoter. Two plasmids were generated: the pX601_ICP0m1-m2 plasmid, which contains the two gRNAs targeting ICP0 (Figure 1B); and the pX601_ICP27m1-m2 plasmid, which contains the two gRNAs targeting ICP27 (Figure 1C). The two plasmids were used to generate two AAV2 vectors, AAV2-SaCas9-ICP0 and AAV2-SaCas9-ICP27.

**Figure 2:**
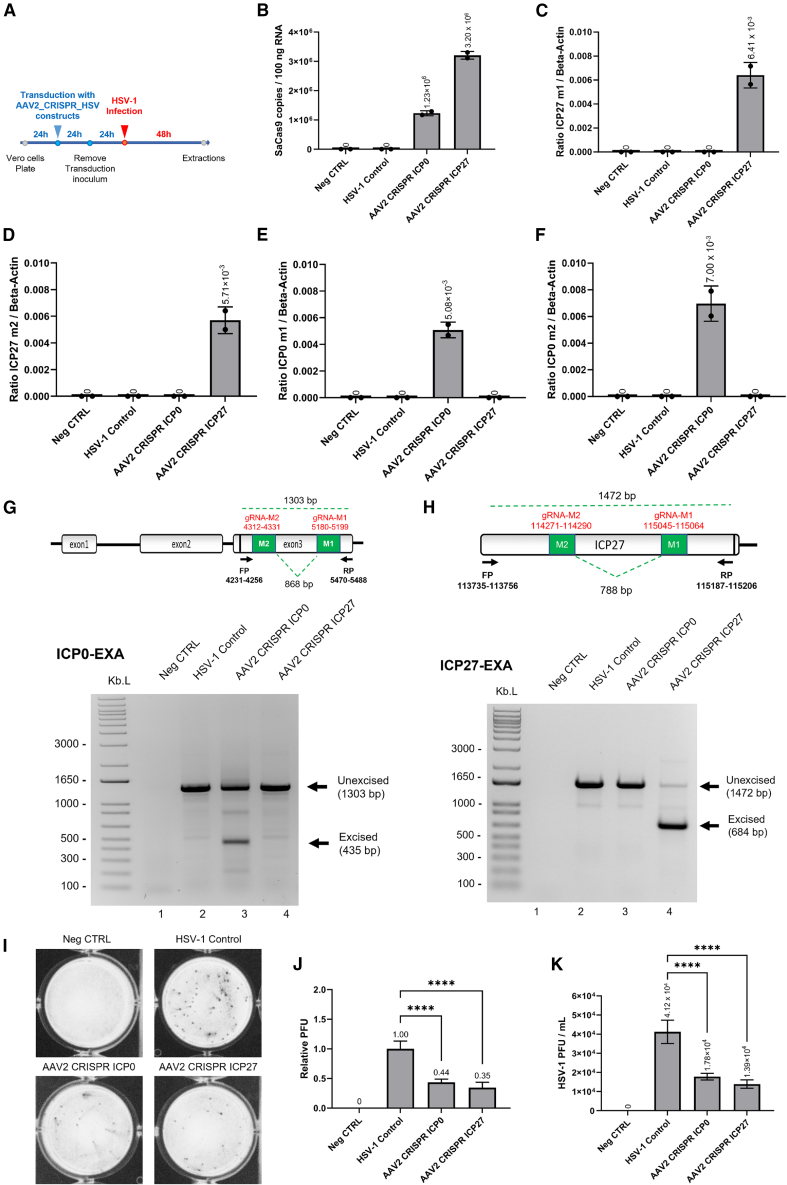
Validation of CRISPR-SaCas9 gene editing platforms in HSV-1-infected Vero cell cultures (A) Schematic timeline of AAV2_CRISPR_HSV-1 construct transductions in Vero cells followed by infection with HSV-1 GFP Patton strain (GenBank MF959544.1https://www.ncbi.nlm.nih.gov/nuccore/MF959544.1). RT-qPCR confirmed (B) SaCas9 expression and the expression of gRNAs ICP27 m1 (C), ICP27 m2 (D), ICP0 m1 (E), and ICP0 m2 (F). Excision assay of HSV-1 ICP0 (G) and ICP27 (H) genes by CRISPR-Cas9 in Vero cells infected with HSV-1 GFP and transduced with AAV2-SaCas9-ICP0 and AAV2-SaCas9-ICP27 vectors, respectively. The gRNAs specifically excised a portion of the targeted genes as shown in lane 4 for ICP27 (G) and lane 3 for ICP0 (H). (I) Representative plaque assay performed on supernatants of Vero cells infected with HSV-1 GFP showed a significant decrease in the number of plaques as result of suppression of ICP0 and ICP27 by CRISPR-SaCas9 in transduced cells. The relative (J) and absolute (K) numbers of plaque-forming units (PFU) were also reported. ∗∗∗∗*p* < 0.0001.

To evaluate the effect of CRISPR-SaCas9 editing platforms on HSV-1 infection, we transduced Vero cells with the two AAV2 vectors. Cells were infected with the HSV-1 GFP Patton strain (HSV-1 GFP; GenBank: MF959544.1) at a multiplicity of infection (MOI) of 1 viral particle per 100,000 cells at 48 h post-AAV2 transduction. Two days post infection, the cells were harvested, and the RNA and gDNA were extracted for analysis (Figure 2A). The expression level of SaCas9 and gRNAs were comparable in cells transduced with the AAV2-SaCas9-ICP0 and AAV2-SaCas9-ICP27 vectors (Figures 2B–2F).

To verify the excision of HSV-1 viral DNA by AAV2-SaCas9 vectors targeting the ICP0 or ICP27 gene, gDNA samples were prepared from the Vero cell experiments and were amplified by PCR using ICP0- or ICP27-specific primers (Table S2). These primers amplify a fragment of 1,472 bp for ICP27 and 1,303 bp for ICP0, whereas the excision of the DNA fragments between the two gRNAs following non-homologous end joining (NHEJ) repair would lead to reduced amplicon size of 435 bp for ICP0 and 684 bp for ICP27, respectively. We observed the excision products at the expected sizes on the agarose gel (Figures 2G and 2H).

In addition to the gDNA analysis, plaque assays were performed on the culture media of the Vero cells to evaluate the effect of CRISPR-Cas9 editing on HSV-1 propagation. As shown in Figures 2I and 2J, both the AAV2-SaCas9-ICP0 and AAV2-SaCas9-ICP27 vectors significantly reduced the HSV-1 viral titer, suggesting a strong antiviral effect.

### Infection and replication of HSV-1 virus in human COs

**Figure 3:**
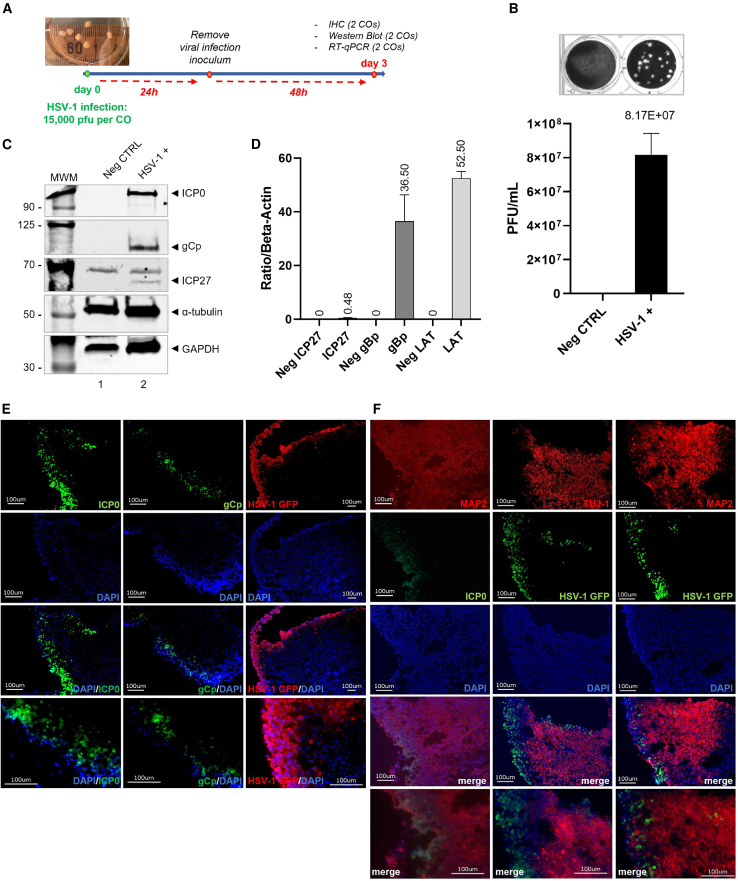
HSV-1 infects and replicates in human COs (A) Schematic timeline of the HSV-1 lytic infection model established in COs. (B) Representative plaque assay from the culture medium of HSV-1 GFP-infected COs. (C) Detection of the viral proteins ICP27, ICP0, and gCp by western blotting. Glyceraldehyde-3-phosphate dehydrogenase (GAPDH) and α-tubulin are shown as internal controls. (D) RT-qPCR of ICP27, gBp, and LAT mRNA levels. IHC on COs for gCp, ICP0, and HSV-1 GFP alone (E) and in co-staining (F) with the neuronal markers MAP2 and class III β-tubulin (TUJ-1).

To establish a 3D *in vitro* culture model of HSV-1 infection, hiPSCs generated from human adult dermal fibroblasts were utilized for the formation of COs. COs were generated as described in the materials and methods and characterized by immunohistochemistry (IHC) staining for neurons, microglia, astrocytes, and oligodendrocytes using anti-MAP2/TUJ-1, anti-IBA1, anti-GFAP, and anti-Olig2 antibody, respectively (Figure S1). Individual COs were infected with 15,000 plaque-forming units (PFU) of HSV-1 GFP. The viral inoculum was removed after 24 h, and the COs were washed in 1× PBS before being transferred to a 24-well ultra-low attachment plate. After 48 h, COs were collected and used for either RNA isolation, protein lysis, or IHC (Figure 3A). Plaque assay was performed on the supernatants of the organoids and the average titer was 8.17 × 10^7^ PFU/mL at 48 h post infection (Figure 3B). Expression of viral proteins ICP0, glycoprotein (gCp), and ICP27 were assessed by western blotting. Consistent with the plaque assay results, all the viral proteins analyzed were expressed, suggesting a productive infection (Figure 3C). In addition, the expression of ICP27, envelope glycoprotein B (gBp), and latency-associated transcript (LAT) were also assessed by RT-qPCR. The expression levels of gBp and LAT were relatively high (Figure 3D), which is consistent with previous findings.^13^ COs infected with HSV-1 were also stained for ICP0, gCp, and GFP (fused in frame to the viral tegument protein US11), and co-stained with the neuronal markers MAP2 and TUJ-1 (Figures 3E and 3F). Uninfected COs were used as negative controls (Figure S2). The ICP0 and GFP proteins were localized on the edge of the organoids, while the neuronal markers MAP2 and TUJ-1 were primarily localized in the center of the COs (Figure 3F). Although it is well known that HSV-1 productively infects neurons, 48 h post infection with HSV-1 has already been in an advanced lytic stage, which is characterized by a massive production of free virions released by exocytosis at the expense of the cellular cytoskeleton structure followed by activation of death signalings.^25^

### Establishment of an HSV-1 latency model in COs

**Figure 4:**
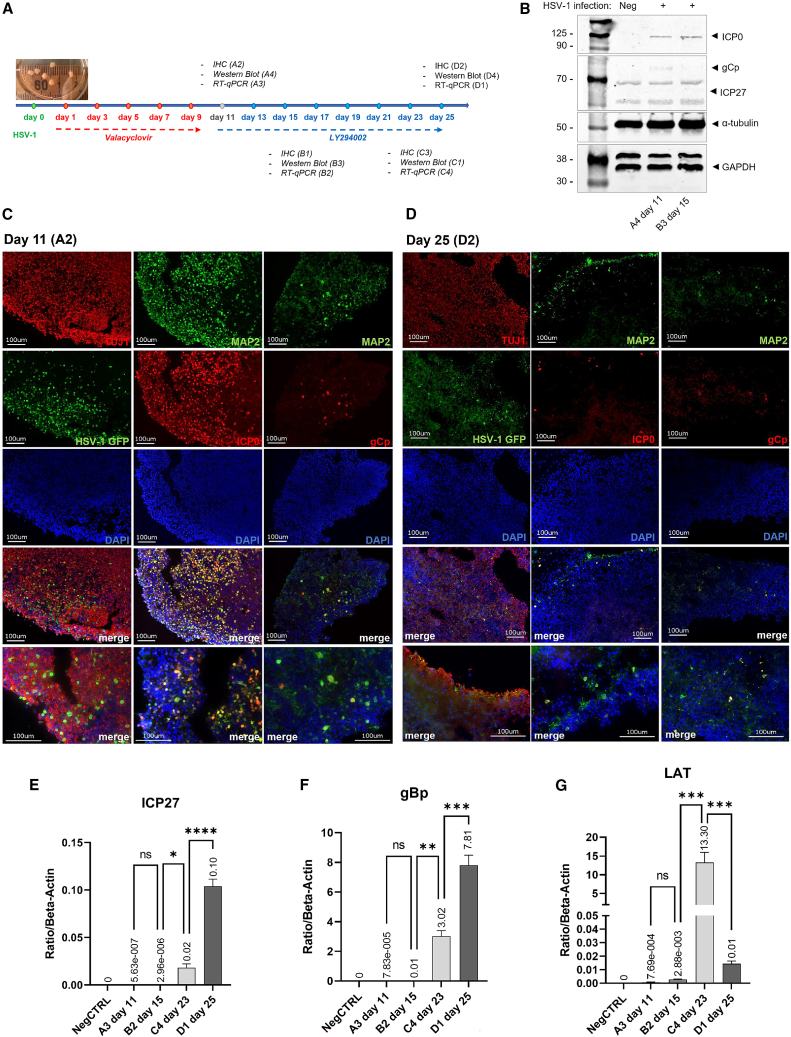
Establishment of HSV-1 latency in COs (A) Schematic timeline of the HSV-1 latency model in COs induced by valacyclovir. (B) Western blot of the viral proteins ICP27, ICP0, and gCp. α-Tubulin and GAPDH proteins were used as internal controls. IHC of COs for gCp, ICP0, and HSV-1 GFP in combination with the neuronal markers MAP2 and TUJ-1 at day 11 (C) and day 25 (D). RT-qPCR of ICP27 (E), gBp (F), and LAT (G) mRNA expression levels at different time points after the induction with LY294002. ∗*p* = 0.0147, ∗∗*p* = 0.001, ∗∗∗ *p* < 0.001, ∗∗∗∗*p* < 0.0001; ns, non-significant.

Once productive/lytic HSV-1 infection was characterized (Figure 3), we next aimed to establish an HSV-1 viral latency model in the COs. Sixteen COs, numbered as A1-A4, B1-B4, C1-C4, and D1-D4, were infected with the HSV-1 GFP virus. At 24 h post infection, the viral inocula were replaced with maturation medium supplemented with 150 μg/mL of valacyclovir per organoid. Valacyclovir is a nucleoside analog, commonly used in the clinic as a primary treatment of HSV-1-associated flare-ups.^14^ As a nucleoside analog metabolized by the viral tyrosine kinase, valacyclovir inhibits viral DNA synthesis and is highly effective in blocking viral replication and lytic infection, inducing the latent state of the virus.^20^ At 11 days post infection, valacyclovir was replaced with 3 μg/mL of LY294002. LY294002 is a phosphatidylinositol 3-kinase inhibitor (PI3Ki) used to induce viral reactivation,^13^ whereas the first group of organoids (A1-A4) were harvested for RNA isolation, protein lysis, and IHC at 11 days post infection before starting with the LY294002 treatment. To induce the reactivation of the virus, the remaining COs were treated with 3 μg/mL of LY294002 every other day starting at day 11. COs were harvested at three different time points: 15 days (B1-B4), 23 days (C1-C4), and 25 days (D1-D4) post infection (Figure 4A).

The plaque assay at 11 days post infection showed an expected viral suppression by valacyclovir (87.5%) (Table 1). Viral reactivation rates with LY294002 treatment were observed in five of eight organoids between days 21 and 25 post infection (Table 1, organoids C1-C4 and D1-D4) and determined as 62.5%. The western blot analysis revealed the expression of ICP0 and gCp at 11 and 15 days post infection. However, the expression of ICP27 was not detected at these time points (Figure 4B). The western blot results were confirmed by IHC staining (Figures 4C, 4D, and S3), which showed a diffused ICP0 expression from the edge to the center of the organoids (Figure 4C). The ICP0 staining largely co-localized with the MAP2 neuronal marker staining, suggesting that HSV-1 productively infected the neurons where it established an unstable latency with leaky expression of the HSV-1 late genes gCp and US11-GFP (Figure 4C). On the other hand, after 4 days of LY294002 treatment (day 15 post infection), a reduced expression of ICP0 and a stable expression of gCp and US11-GFP were observed (Figure S3). At day 25, a more robust release of HSV-1 virions in the supernatant was observed (Table 1), while only US11-GFP was primarily detectable in neurons (Figure 4D), indicating that the HSV-1 virus was reactivated from its latency. The expression levels of ICP27 (Figure 4E) and gBp (Figure 4F) were low at days 11 and 13, and then elevated at days 23 and 25. The LAT expression level peaked at day 23, and then rapidly decreased at day 25 (*p* = 0.0003) (Figure 4G), suggesting viral reactivation. Overall, these observations suggest that HSV-1 can form latency in COs, making it a suitable *in vitro* model for testing the efficacy of new therapeutic regiments.

**Table 1:** Plaque assay results from CO culture medium collected at different time points during the latency induced by valacyclovir

	Organoids (PFU/mL)																
Time points	A1	A2	A3	A4	B1	B2	B3	B4	C1	C2	C3	C4	D1	D2	D3	D4	percentage viral suppression by valacyclovir: 87.5 (day 11)
d3	2.50E+02	0	0	0	5.00E+02	0	0	0	0	0	0	0	0	0	0	0	
d5	0	0	0	0	0	0	0	0	0	0	0	0	0	0	0	0	
d7	0	0	0	0	0	0	0	0	0	0	0	0	0	0	0	0	
d9	5.00E+01	0	0	0	5.00E+03	0	0	0	0	0	0	0	0	0	0	4.00E+04	
d11	0	0	0	0	3.50E+04	0	0	0	0	0	0	0	0	0	0	5.00E+02	
d13	n.a.	n.a.	n.a.	n.a.	3.50E+03	0	0	1.00E+02	0	5.00E+01	0	0	5.00E+02	0	0	1.00E+03	percentage reactivation: 62.5 (days 21–25)
d15	n.a.	n.a.	n.a.	n.a.	1.50E+03	0	0	5.00E+03	5.00E+01	0	0	0	0	0	0	5.00E+02	
d17	n.a.	n.a.	n.a.	n.a.	n.a.	n.a.	n.a.	n.a.	1.00E+02	2.50E+02	0	5.00E+01	0	0	0	0	
d19	n.a.	n.a.	n.a.	n.a.	n.a.	n.a.	n.a.	n.a.	2.50E+02	5.00E+01	0	0	0	0	0	0	
d21	n.a.	n.a.	n.a.	n.a.	n.a.	n.a.	n.a.	n.a.	1.00E+03	3.00E+03	0	0	0	0	0	0	
d23	n.a.	n.a.	n.a.	n.a.	n.a.	n.a.	n.a.	n.a.	5.00E+03	5.00E+03	0	5.00E+02	1.50E+03	0	0	0	
d25	n.a.	n.a.	n.a.	n.a.	n.a.	n.a.	n.a.	n.a.	n.a.	n.a.	n.a.	n.a.	8.00E+03	2.00E+03	0	0	

n.a., not applicable.

### Suppression of HSV-1 reactivation and replication by CRISPR-SaCas9 gene editing in COs

**Figure 5:**
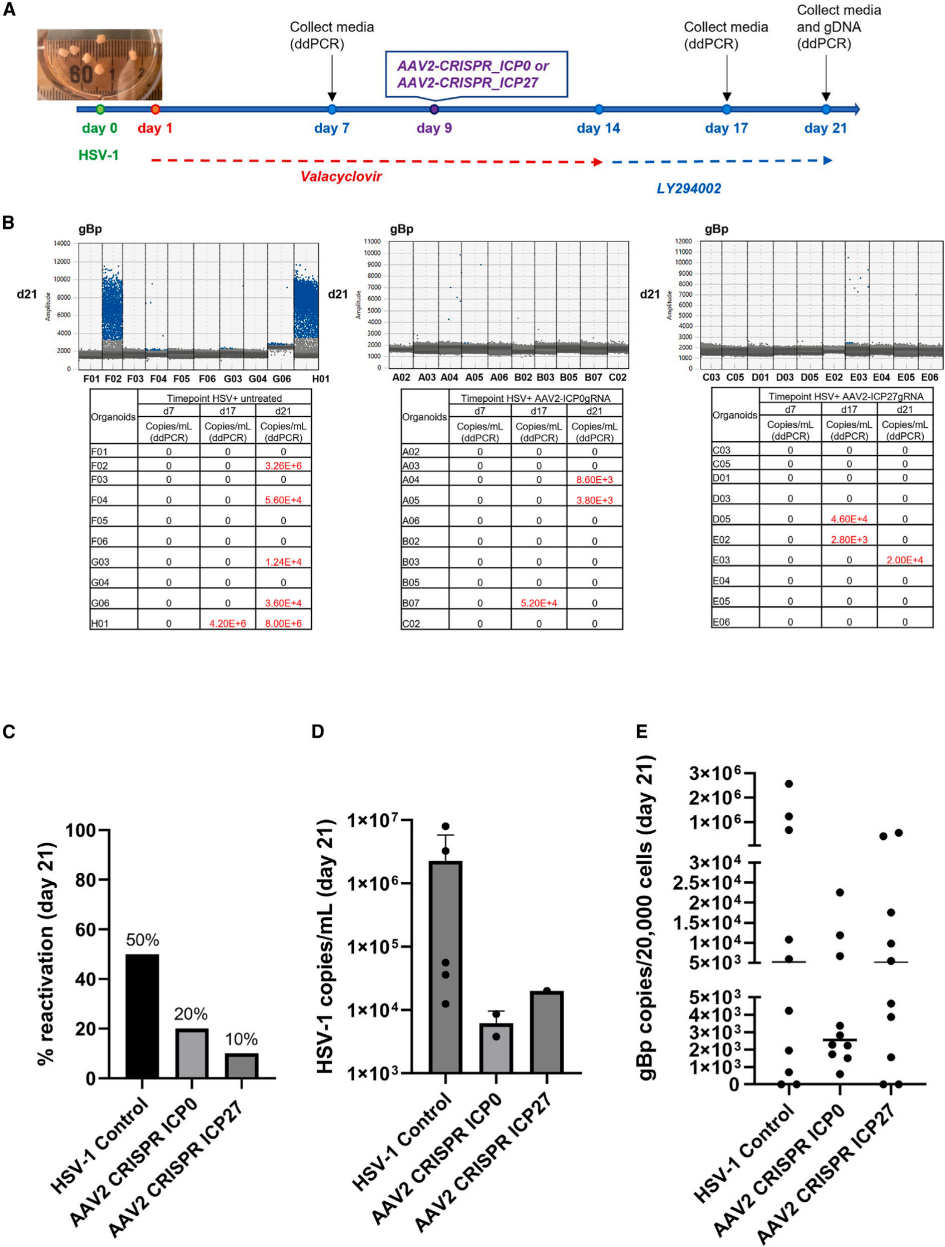
Suppression of HSV-1 reactivation and replication by CRISPR-SaCas9 gene editing platforms in COs (A) Schematic timeline of COs infected with HSV-1 GFP and transiently transduced with AAV2-SaCas9-ICP0 or AAV2-SaCas9-ICP27 vector. (B) ddPCR analysis of HSV-1 genome copy numbers in CO culture medium collected at days 7, 17, and 21. The tables show the raw viral DNA copy numbers from the untreated, AAV2-SaCas9-ICP0, and AAV2-SaCas9-ICP27 groups, respectively. Representative ddPCR plots are shown above each table. (C) Percentage of viral reactivations in the control and AAV2-transduced COs at day 21. (D and E) ddPCR analysis of the HSV-1 viral DNA in the culture medium (D) and in the genomic DNA isolated from the COs (E) at day 21.

The HSV-1 latency model in COs described above was used to evaluate the efficacy of the AAV2-SaCas9-ICP0 and AAV2-SaCas9-ICP27 vectors. Thirty COs infected with HSV-1 GFP virus were treated daily with valacyclovir for 8 days, starting at 24 h post HSV infection (Figure 5A). COs were then either transduced with the AAV2-SaCas9-ICP0 or AAV2-SaCas9-ICP27 vector or left untreated at 9 days post infection. At 14 days post infection, the valacyclovir was replaced with LY294002, and the COs were treated every other day for 7 days. The culture media were collected from individual organoids at days 7 and 17 to assess the viral load by droplet digital PCR (ddPCR). The culture media and gDNA samples were collected from all 30 organoids at day 21. As shown in Figures 5B and 5C, the HSV-1 reactivation rate was 50% in untreated control organoids at day 21, whereas the reactivation rates in the organoids treated with AAV2-SaCas9-ICP0 and AAV2-SaCas9-ICP27 were only 20% and 10% at day 21, respectively. At day 21, the HSV-1 viral load in the supernatants from untreated organoids was 3 log higher than in the supernatants from organoids treated with AAV2-SaCas9-ICP0 or AAV2-SaCas9-ICP27 (Figure 5D). Moreover, the organoids treated with AAV2-SaCas9-ICP0 or AAV2-SaCas9-ICP27 showed a reduction in the HSV-1 viral load in the cell lysate compared with untreated organoids, as measured by ddPCR using a primer/probe set targeting the HSV-1 gBp gene. (Figure 5E). Additional information about the ddPCR plots of the HSV-1 DNA copies in the supernatants and gDNA samples of COs are shown in Figure S4. Excision of the HSV-1 genome by paired ICP0 or ICP27 gRNAs was also analyzed by PCR assays (Figure S5). The ICP0 and ICP27 viral genome excision bands were observed in 9/10 and 8/10 COs treated with AAV2-SaCas9-ICP27 and AAV2-SaCas9-ICP0, respectively (Figure S5). In summary, our data suggest that the CRISPR-Cas9 gene editing treatment effectively suppresses HSV-1 reactivation in COs.

## Discussion

Progress in developing therapeutic strategies toward the elimination of latent HSV-1 infection and the treatment of associated disease complications remain an unmet public health concern. Current treatment regimens for HSV-1 primary infection or reactivation are not specific and do not prevent the establishment of latent viral infection or reactivation.^14^ The CRISPR-Cas9 genome editing strategy could be a potential tool for targeting not only the actively replicating virus but also eliminating the latent viral reservoirs. Here, we used the CRISPR-Cas9 platform to target two regions within two HSV-1 immediate-early genes, ICP0 and ICP27, which are required for viral DNA replication, transcription, and regulation of both viral and cellular gene expressions. The simultaneous expression of SaCas9 and gRNAs targeting HSV-1 ICP0 or ICP27 genes in Vero cells infected with HSV-1 showed specific deletions in the target genes. This multiplex gRNA approach allowed the excision of the large DNA fragment from the viral genome, preventing the development of escape mutant viruses. In fact, previous studies have shown that single gRNAs led to viral escape and CRISPR-Cas resistance due to the cellular NHEJ repairing of viral DNA.^26^

For efficient delivery of our Cas9 and gRNAs complexes, we used AAV2 as a gene delivery vehicle due to its high neurotropic nature^27^^,^^28^ and promising application in clinical trials.^29^ Different AAV serotypes show distinct cell and tissue tropisms.^29^ Results from our AAV2 transduction experiments in Vero cells demonstrated high excision efficiency of target genes and strong reduction in HSV-1 viral replication.

To generate an *in vivo* comparable model, we have established COs from hiPSCs that were generated in our laboratory from adult dermal fibroblasts. Before the infection with HSV-1, COs were characterized by IHC for brain cell types including neurons, microglia, astrocytes, and oligodendrocytes using MAP2/TUJ-1-, IBA1-, GFAP-, and Olig2-specific antibodies, respectively. From the IHCs, it emerged that neurons, microglia, astrocytes, and oligodendrocytes are well represented between 60 and 80 days after the induction of embryoid bodies (EBs). In acute HSV-1 infection (48 h post infection), expression of viral proteins ICP0, gCp, and ICP27 were assessed by western blot and all three genes were expressed. In addition, expressions of immediate-early protein ICP27, envelope gBp, and LAT were also confirmed by RT-qPCR as observed in another similar study.^13^ The elevated levels of the LAT transcript detected at 48 h acute infection likely results from the LAT primary transcript, which tends to accumulate significantly during the later stages as a gamma transcript in productive infections across various cell types.^30^ A co-staining of viral protein ICP0, glycoprotein C (gCp), and HSV-1 GFP with neuronal markers MAP2/TUJ-1 suggested the localization of these proteins on the edges of the organoids at 48 h acute infections, whereas the neuronal markers localized mostly in the cores of COs. On the other hand, when we established the HSV-1 latency model in COs, ICP0 expression was spread from the outer surface of the organoids and reached to the center core, suggesting that COs may serve as an *in vitro* platform to study HSV-1 trafficking through complex neuronal tissue structures.

To determine if COs could be a model for establishing HSV-1 latency and platform for testing anti-HSV-1 modalities including CRISPR-Cas9 gene editing approaches, they were tested for viral latency establishment and rebound capabilities. Interestingly, approximately 12.5% of the organoids showed spontaneous viral reactivation at 11 days post infection, which is in line with the 17% reactivation rate observed by D’Aiuto et al.^13^ between 8 and 11 days post infection upon antiviral removal. On the other hand, we observed 62.5% viral reactivation rates induced by LY294002 only after 10 days of treatments. These data are consistent with the establishment of an HSV-1 latency model in COs that could be applied for assessing the efficacy of new therapeutic regimens.

During the past decade, several studies have been conducted in cell lines (Vero cells mainly) and primary cultures (fibroblasts and neuronal cells) to test the efficacy and the safety of HSV1-specific CRISPR-Cas9. Successful editing of the HSV-1 genome in gE, thymidine kinase (UL23), ICP0, ICP4 UL29, UL52, and UL8 genes by SpCas9 and SaCas9 has been shown recently.^15^^,^^19^^,^^22^^,^^31^^,^^32^ Moreover, Oh et al. investigated for the first time the mechanism of how CRISPR-Cas9 cleaved the genome of the replication-defective HSV-1 d109 virus, which showed heterochromatin loading in viral DNA mimicking the latent state of the viral HSV-1 genome.^20^ Experiments with this quiescence infection model indicated that anti-HSV-1 gRNAs targeting four viral essential genes (UL29, UL30, UL54/ICP27, and ICP4) could reduce reactivation of quiescent HSV-1 d109 genomes.^20^ Overall, these studies showed many limitations and safety issues related to the gene editing system and the selection of delivery methods. Dual targeting of HSV-1 DNA regions resulted in a more efficient genome editing than targeting a single site. Dual targeting generates multiple cleavages on target DNA, leading to a more effective deletion or mutation of the target gene. Moreover, HSV-1 DNA in latent state appears to have a more compacted chromatin structure than the replicating viral DNA. CRISPR-Cas9 can edit both non-replicating and replicating genomes, although it mutates the replicating genomes much more efficiently.^33^^,^^34^^,^^35^ Therefore, improving the access of CRISPR-Cas9 to HSV-1 latent genome target sites may increase the editing efficacy and enhance the reduction of HSV-1 latent infection.

Finally, the delivery strategy is crucial in the achievement of the final goal. Currently, AAV and lentiviruses are the most used viral vector candidates. Considering the limited gene-carrying capacity of AAV vectors, utilizing lentiviral vectors to deliver CRISPR elements may represent the best approach to deliver Cas9 and gRNAs. However, lentivirus vectors can potentially integrate the carrying genome in the host gDNA and potentiates off-target effects, whereas AAV constructs will likely persist for the short term in dividing cells and extended time in non-dividing cells such as neurons and may require a stronger promoter for an efficient nuclease expression.^36^^,^^37^

In our study, we have taken into account all the limitations of the latency model systems, CRISPR-Cas9 editing approaches, and delivery platforms, and balanced them with their strengths. We have established a new *in vitro* 3D model of HSV-1 lytic and latent infection in COs to test the promising CRISPR-SaCas9 gene editing strategy on resting and actively replicating genome of HSV-1. Our results revealed a reduction in HSV-1 reactivation rates in COs treated with AAV2_CRISPR_ICP0 and AAV2_CRISPR_ICP27 than untreated controls. 3D COs are clearly an improved *in vitro* platform over 2D cell cultures with their tissue-cell complexities comparable with *in vivo* conditions.^38^ Moreover, these models can be utilized for testing therapeutic strategies, considering their more stable genetic background derived from re-programmed primary human fibroblasts rather than genomically unstable immortalized cell lines.

In summary, our results demonstrated that targeting multiple target sites in the immediate-early genes of HSV-1 with CRISPR-Cas9 is capable of introducing edits and large deletions on target genes, suggesting that a multiplex gRNA approach may be considered as a therapeutic tool to eliminate HSV-1 gDNA from latently infected cells.

## Materials and methods

### Cell culture and COs

Vero cells were grown in DMEM supplemented with 10% FBS as we have described previously.^22^ COs were generated from hiPSCs, originated from human dermal fibroblasts, following the protocol provided with the STEMdiff COs Kit (STEMCELL Technologies, no. 08570) and the STEMdiff CO Maturation Kit (STEMCELL Technologies, no. 08571). In brief, hiPSCs were harvested, gently resuspended by pipetting, and plated in a 96-well round-bottom ultra-low attachment plate at the confluency of 9,000 cells/well. The 96-well plate was incubated at 37°C for 24 h and the next day small EBs (100–200 μm) were observed with a layer of unincorporated cells around the central EB. When EBs reached a diameter of 400–600 μm and exhibited round and smooth edges (usually 5 days), they were thus transferred in a 24-well ultra-low attachment plate containing 500 μL of induction medium. After 48 h of induction at 37°C, EBs maintained smooth edges and developed optically translucent edges. At this stage, each EB was coated with Matrigel (Corning, no. 354277) and dropped in a 6-well ultra-low adherent plate containing 3 mL of expansion medium. After 3 days of incubation at 37°C, embedded organoids developed expanded neuroepithelia as demonstrated by budding of the EB surface. At this stage of maturation, the organoids were placed on an orbital shaker at a speed of 70 rpm in a 37°C incubator, and the expansion medium was replaced by the maturation medium (STEMCELL Technologies, no. 08571).

### HSV-1 ICP0 and ICP27 gRNA design and pX601_SaCas9_gRNA(s)_plasmids

The genomic sequences of HSV-1 ICP0 and ICP27 were obtained from the NCBI database (RefSeq NC_001806.2, https://www.ncbi.nlm.nih.gov/nuccor/NC_001806.2). Two gRNAs for each viral gene were designed and selected using the Benchling online platform (https://www.benchling.com/) (Table S1). A pair of DNA oligos from each target sequence were designed in forward and reverse orientations (Table S3), based on published and recommended flanking sequences for the vector pX601-AAV-CMV::NLS-SaCas9-NLS-3xHA-bGHpA; U6::BsaI-sgRNA (pX601)^39^^,^^40^ (Addgene, plasmid no. 61591). Each pair was annealed in a thermocycler at 95°C for 5 min and ramped from 95°C to 25°C in 45 min, using 5 μL of each oligo at the concentration of 10 μM in presence of T4 DNA ligase buffer in 20 μL of PCR water. Annealed oligo pairs were then cloned into BsaI linearized px601 to generate pX601_ICP0m1, pX601_ICP0m2, pX601_ICP27m1, and pX601_ICP27_m2. The insertion of the gRNAs was confirmed by Sanger sequencing and then each U6::gRNA cassette was cloned in tandem using the In-Fusion HD Cloning Plus Kit (Takara Bio, no. 638910) and specific primers flanking the U6::gRNA cassette (Table S3). Two plasmids were generated by In-Fusion HD cloning: (1) pX601_ICP0m1-m2 contains the two gRNAs targeting ICP0 (m1 and m2); (2) pX601_ICP27m1-m2 contains the two gRNAs targeting ICP27 (m1 and m2). Plasmid preps were prepared using a Plasmid Maxi kit (QIAGEN, no. 12162). The plasmid pX601_ICP0m1-m2 and pX601_ICP27m1-m2 were then sent to the University of North Carolina (UNC) Vector Core for AAV2 Custom Vector Production. The final titer yield of the two vectors (AAV2_CRISPR_ICP0 and AAV2_CRISPR_ICP27) was ∼1–4 × 10^12^ vg/mL. The Benchling online platform was also used to determine the off-target sequences and scores.

### Infection of Vero cells and COs with HSV-1

Forty-eight hours after transduction with AAV2 vectors, Vero cells were infected with the synthetic HSV-1 GFP-US11 strain Patton (HSV-1 GFP) at MOI of 1 × 10^−5^ (GenBank: MF959544.1, https://www.ncbi.nlm.nih.gov/nuccore/MF959544.1/). HSV-1 GFP is a recombinant virus that expresses enhanced green fluorescent protein fused in-frame to the viral tegument protein US11. The GFP-US11 fusion protein is expressed at the true-late stage of viral cycle and accumulates at a sufficient level for detection by light microscopy after the onset of viral DNA replication. This provides an easily scored indicator of acute infection and reactivation of HSV-1, replicating with the same efficiency and kinetics as the parental wild-type Patton virus.^41^^,^^42^ In brief, the viral inoculum was prepared in optiMEM and the volume was adjusted to the minimal amount required to cover the cellular monolayer. After 2 h of incubation at 37 °C, the viral inoculum was removed and fresh DMEM supplemented with 10% FBS was added back on. Cells were then incubated for 2 days before being harvested for RNA and DNA extraction and supernatants were collected to perform the plaques assays. COs were infected in a 96-well plate with 15,000 PFU of HSV-1 GFP in 200 μL of maturation medium. The viral inoculum was removed after 24 h, the COs were washed in 1× PBS and cultured in 1 mL of maturation medium per well in a 24-well ultra-low attachment plate. For the HSV-1 acute infection experiment, the COs were harvested 48 h post infection. For HSV-1 latency experiments, after infection with 30,000 PFU of HSV-1 GFP, the viral inoculum was replaced with 1 mL of maturation medium supplemented with 150 μg of valacyclovir per organoid. Culture medium with 150 μg/mL of valacyclovir was changed bi-daily until 11 days post infection, when valacyclovir was replaced with 3 μg/mL LY294002 (Sigma-Aldrich, no. 440204). LY294002 is a PI3Ki used to induce viral reactivation.^13^ COs were treated with LY294002 bi-daily for 14 days (until 25 days post infection) when the experiments were ended. For AAV2_CRISPR_ICP0 and AAV2_CRISPR_ICP27 treatment experiments, COs were infected in 96-well plate with 60,000 PFU of HSV-1 GFP in 200 μL of CO maturation medium. Next day, the viral inocula were replaced with 200 μL of maturation medium supplemented with valacyclovir. The culture media were changed bi-daily with fresh maturation medium containing valacyclovir until 9 days post infection, when the COs where transduced with the AVV2 vectors. COs were treated with valacyclovir for an additional 4 days after AAV2 transductions. At 14 days post infection, valacyclovir treatment was replaced with 3 μg/mL LY294002. COs were treated with LY294002 bi-daily for an additional 7 days (until 21 days post infection).

### HSV-1 plaque assay

Supernatant from Vero cells and COs containing newly generated viral particles were collected and titered as PFU by infecting Vero cells (plated at 100% confluency in 48-well plates) with serial dilutions of the supernatant. At 48 h post infection, cells were fixed with 10% trichloroacetic acid (Sigma-Aldrich, no. T0699-100ML) for 10 min and then stained with 0.05% of Crystal violet (Sigma-Aldrich, no. C0775-25G) for 15 min. The plaques were counted and graphed using GraphPad Prism version 9.4.1.

### AAV2_CRISPR vector transduction of Vero cells and COs

Vero cells were plated at 60% confluency the day before the AAV2 vector transductions. AAV2_CRISPR_ICP0 and AAV2_CRISPR_ICP27 inocula were prepared at MOI of 250,000 for each vector in a minimal volume of optiMEM, adjusted to cover the cellular monolayer. AAV2 particles were left absorbed by cells for at least 2 h in an incubator at 37°C on an orbital shaker at a speed of 100 rpm. After 2 h, fresh culture medium with serum was added without removing the AAV inoculum and the cells were incubated overnight at 37°C. Next day, the medium containing the AAV inoculum was removed and fresh culture medium with serum was added. After 48 h, the thus transduced cells were infected with HSV-1 GFP. COs were transduced at 9 days post infection with AAV2_CRISPR_ICP0 and AAV2_CRISPR_ICP27 at MOI of 400,000 vGCs per organoid. In a 24-well plate, 250 μL of optiMEM containing the AAV2 particles was added to the organoids, and they were left absorbed for at least 7 h in an incubator at 37°C on an orbital shaker at a speed of 75 rpm. After 7 h, fresh maturation medium was added to each organoid up to 500 μL without removing the 250 μL of AAV inoculum and the thus transduced COs were incubated overnight at 37°C. Next day, the inoculum with the AVV2 particles was removed and the COs were treated with valacyclovir.

### Cryostat sectioning of COs

HSV-1-infected and uninfected COs from experiments of HSV-1 acute infection and latency models were embedded in an optical cutting temperature (O.C.T.) matrix (Azer Scientific, no. ES34821) designed for cryostat sectioning. In brief, COs were transferred into 50 mL conical tube and washed for 10 min with 5 mL of 1× PBS 3 times at room temperature (RT). After washing, COs were incubated for 16 h at 4°C in 5 mL of 4% paraformaldehyde in 1× PBS. Then COs were washed 10 min with 5 mL of 0.1% Tween in 1× PBS (PBST) 3 times and incubated overnight in 1× PBS. COs were then incubated at 4°C in 5 mL of 30% sucrose in 1× PBS solution for 24–48 h, transferred to a labeled Cryomold (Tissue-Tek Cryomold Biopsy, no. 4565), embedded in the O.C.T. matrix, and snap-frozen in a mixture of 100% ethanol and dry ice. The COs blocks were sectioned in 10 μm slides using a cryostat at 16°C–18°C. COs sections were adhered to super frost-charged glass slides and kept at RT for 20 min before staining procedures.

### Immunofluorescence staining of COs

CO sections were washed with 0.05% Tween 20 in 1× PBS (PBST) for 5 min and then a hydrophobic barrier was created with ImmEdge Pen (Vector, no. H-4000). They were permeabilized with 0.3% TX-100 in 1× PBS containing 1% normal serum of secondary antibody’s host for 15 min at RT and then washed 5 min with 0.05% PBST 3 times. After 1 h blocking with 5% normal serum of secondary antibody’s host at RT in a humidified chamber, primary antibody was diluted in Dako Antibody Diluent (Agilent Technologies, no. S0809) and incubated overnight at 4°C. Next day, the sections were washed for 5 min with 0.05% PBST 3 times and secondary antibody was diluted in Dako Antibody Diluent and incubated for 1 h at RT. After incubation, excess antibodies were washed for 5 min with 0.05% PBST 3 times, 5 min with 1× PBS, and 5 min with DI water. The coverslip was applied by using the ProLong Gold antifade reagent with DAPI (Invitrogen, no. P36935). The slides were imaged after 30 min by fluorescence microscopy.

### Antibodies

The following antibodies were used for the immunofluorescence staining of COs: mouse monoclonal α-ICP0 HSV-1 (11060) (1:50, Santa Cruz, no. sc-53070); mouse monoclonal α-gC HSV-1 (3G9) (1:400, Abcam, no. ab6509); rabbit polyclonal α-GFP (living color) (1:200, Takara, no. 632592); mouse monoclonal α-tubulin beta III human (TUJ-1) (1:300, Santa Cruz, no. sc-58888); rabbit monoclonal α-MAP2 human (D5G1) (1:500, Cell Signaling, no. 8707S); rabbit polyclonal α-Olig2 human (1:1,000, Abcam, no. ab254043); mouse monoclonal α-GFAP human (GA5) (1:50, Millipore, no. MAB360); and rabbit polyclonal α-IBA1 human (1:1,000, FUJIFILM Wako Pure Chemicals, no. 19741). Alexa Fluor 488 donkey anti-mouse IgG (H+L) (1:1,000, Invitrogen, no. A21202) and Alexa Fluor 594 donkey anti-rabbit IgG (H+L) (1:1,000, Invitrogen, no. A21207) were used as secondary antibodies. The following antibodies were used for the western blot: mouse monoclonal α-ICP0 HSV-1 (11060) (1:1,000, Santa Cruz, no. sc-53070); mouse monoclonal α-gC HSV-1 (3G9) (1:1,000, Abcam, no. ab6509); mouse monoclonal α-ICP27 HSV-1/2 (H1113) (1:1,000, Santa Cruz, no. sc-69807); mouse monoclonal anti-α-tubulin (B7) antibody (1:5,000, Santa Cruz, no. sc-5286); and mouse monoclonal α-GAPDH (6C5) (1:1,000, Santa Cruz, no. sc-32233). IRDye goat anti-mouse 800CW (LI-COR, no. 926-32210) and IRDye goat anti-mouse 680RD (LI-COR, no. 926-68070) were used as secondary antibodies.

### Retro-transcription quantitative PCR

To determine the expression of gRNAs and SaCas9 after AAV2 vector transduction in Vero cells, RT-qPCR was performed. In brief, RNA was extracted from Vero cells using the Monarch Total RNA Miniprep Kit (New England BioLabs, no. T2010S) following the manufacturer’s instructions. One microgram of RNA was retrotranscribed using the M-MLV Reverse Transcriptase (200 U/μL) kit (Invitrogen, no. 28025013) in the presence of 1 μL of dNTPs mix, 10 mM each (Invitrogen, no. 18427013), 1 μL of 40,000 U/mL murine RNase Inhibitor (New England BioLabs, no. M0314S), 2 pmol of gRNA scaffold reverse primers (RT_gRNA_px60_scaffold_Rev: 5′-CGC CAA GTT GAC GAG ATA A-3′), and 2 μL of 60 μM of Random Primer Mix (New England BioLabs, no. S1330S). One hundred nanograms of the thus obtained cDNA was used in qPCR of gRNA and SaCas9 expression. For gRNA qPCR, a universal reverse primer containing the FAM probe (Table S4) at the final concentration of 0.2 μM was used in 20 μL mixture of Luna Universal Probe qPCR Master Mix (New England BioLabs, no. M3004) with gRNA-specific primers used at a final concentration of 0.4 μM (Table S4). Forty cycles of two-step amplification (one step at 95°C for 15 s followed by one step of 30 s at 60°C including the plate read) were performed with one preliminary denaturation step of 1 min at 95°C. For SaCas9 qPCR, 100 ng of extracted RNA was directly used in 20 μL mixture of Luna Universal Probe One-Step RT-qPCR Kit Protocol (New England BioLabs, no. E3006), containing SaCas9-specific primers and FAM probe used at the final concentration of 0.4 and 0.2 μM, respectively (Table S4). An absolute quantification of SaCas9 was performed using a standard curve constructed on serial dilutions (range 1.027 × 10^0^ to 1.027 × 10^10^) of px601 vector without gRNAs. Forty cycles of two-step amplification (one step at 95°C for 15 s followed by one step of 30 s at 60°C including the plate read) were performed with one preliminary reverse transcription at 55°C for 10 min followed by a denaturation step of 1 min at 95°C. The *Macaca mulatta* β-actin was used as internal control in the RT-qPCR of RNA extracted from Vero cells. To determine the expression of HSV-1 gBp, ICP27 and LAT in COs infected with HSV-1 GFP, 500 μg of RNA was retrotranscribed using M-MLV Reverse Transcriptase (200 U/μL) kit (Invitrogen, no. 28025013) in presence of 1 μL of dNTPsmix, 10 mM each (Invitrogen, no. 18427013), 1 μL of 40,000 U/mL murine RNase Inhibitor (New England BioLabs, no. M0314S), 20 pmol of LAT Intron reverse primers (LAT_Intron_cDNA_Rev: 5′-GTG GTC GGA CGG GTA AGT AA-3′)^13^ for LAT, and 1 μL of Oligo d(T)18 mRNA Primer (New England BioLabs, no. S1316S) for gBp, ICP27, and internal control (Table S5). Fifty nanograms of the thus obtained cDNA was used in qPCR for HSV-1 gBp, ICP27, and LAT,^13^ using specific primers (Table S5) at a final concentration of 0.4 μM in 20 μL mixture of TB Green Premix Ex Taq II (2×) (Tli RNaseH Plus), Bulk (Takara, cat. no. RR820L). Forty-five cycles of two-step amplification (one step at 95°C for 5 s followed by one step of 30 s at 60°C including the plate read) were performed with one preliminary denaturation step of 30 s at 95°C and a one final melting step. Human β-actin gene was used as internal control (Table S5). All the qPCRs were run with the Light Cycler 96 System by Roche.

### Excision assays

An excision assay was performed on HSV-1 ICP0 and ICP27 sequences from Vero cells and COs infected with HSV-1 and transduced by AAV2_CRISPR constructs. Total gDNA was isolated from cells using a gDNA purification kit according to the manufacturer’s instructions (NucleoSpin Tissue by Macherey-Nagel, no. 740952), and the targeted regions of the ICP0 and ICP27 genes were amplified by PCR using flanking primers (Table S2). The PCR reactions were performed in 50 μL containing 250 ng of gDNA, 2.5 units of FailSafe Enzyme mix (BIOSEARCH Technologies, no. E0030-2.5D3), FailSafe PCR 2× PreMix J (BIOSEARCH Technologies, no. SS000157-D2), and specific primers at a final concentration of 500 nM. The PCR products were run on 1% agarose gel and the expected unexcised fragments were 1,472 and 1,303 bp for ICP27 and ICP0, respectively, whereas the expected excised fragments were of 684 and 435 bp for ICP27 and ICP0, respectively.

### Western blotting

Before performing any western blot assay, whole-cell extracts were prepared in Pierce RIPA buffer (Thermo Scientific, no. 89900) containing mammalian protease inhibitor cocktail (Sigma-Aldrich, no. P8340-1ML). The protein concentration of the lysates was calculated using Bradford solution (Bio-Rad, no. 500-0006). Protein lysates were separated by SDS-PAGE into a 10% polyacrylamide gel at 110 V for 1 h, transferred to nitrocellulose membranes (0.45 μm) at 50 mA overnight, and immunoblotted with primary antibodies 1/1,000 diluted in 2.5% milk in 1× PBST for 3 h at RT (with the exception of anti-α-tubulin antibody incubated for 1 h at RT) followed by infrared (IRDye) secondary antibodies (1/5,000 diluted) for 1 h at RT (see above antibodies). The membranes were then washed 3 times for 10 min with 1× PBST solution and scanned with the Odyssey CLx Imaging System (LI-COR, Lincoln, NE). Finally, the images were acquired by the LI-COR Odyssey software and the intensity of each band was quantified using the ImageJ software (National Institutes of Health) and intensities normalized using α-tubulin or GAPDH as equal loading controls.

### Quantification of HSV-1 GFP DNA by ddPCR

A duplex ddPCR was performed to measure the levels of cellular HSV DNA using the QX200 droplet digital PCR system (Bio-Rad Laboratories, Hercules, CA), as described previously.^43^ In brief, DNA samples were extracted from COs using a gDNA purification kit according to the manufacturer’s instructions (NucleoSpin Tissue by Macherey-Nagel, no. 740952). Copies of HSV DNA were quantified using specific HSV-1 gBp primers and FAM probe (Table S5). Cell numbers were determined using specific human TERT primers and HEX probe. Human TERT is a housekeeping gene for cell count that exists at two copies/cell. The ddPCR reaction mixture consisted of 12.5 μL of 2× ddPCR Supermix for Probes (No dUTP) (Bio-Rad, no. 1863024), 1.25 μL of each 20× primer and probe mix for a final concentration of 500 nM, and 10 μL of template DNA from 1/200 dilution of 50 μL of supernatant or 600 pg of gDNA in a final volume of 25 μL. Twenty microliters of each reaction mixture was loaded onto a disposable plastic cartridge (DG8 Cartridges and Gaskets, Bio-Rad, no. 1864007) with 70 μL of droplet generation oil (Bio-Rad, no. 1863005) and placed in the droplet generator (Bio-Rad Laboratories). The droplets generated from each sample were transferred to a 96-well PCR plate, and PCR amplification was performed with the following conditions: 95°C for 10 min, 40 cycles of 94°C for 30 s, and 60°C for 1 min, followed by 98°C for 10 min and ending at 4°C. After amplification, the plate was loaded onto the droplet reader (Bio-Rad Laboratories). Data were analyzed with QuantaSoft analysis software (Bio-Rad) and quantitation of target molecules presented as copies/mL or copies/20,000 cells. For quantification of cellular HSV levels, values were normalized to cellular TERT levels.

### Statistical analysis

All the values on the graphs are presented as mean and the standard deviations are reported as error bars on the histograms. Student’s t test and ANOVA were performed and *p* values <0.05 were considered statistically significant. The raw data were analyzed by GraphPad Prism version 9.4.1.

## Data and code availability

All data in this manuscript are available from the authors on reasonable request.

## Supplemental information

Document S1. Figures S1–S5 and Tables S1–S5

Document S2. Article plus supplemental information
