# Supplementary material for: Suppression of HSV-1 infection and viral reactivation by CRISPR-Cas9 gene editing in 2D and 3D culture models
Source: Mol Ther Nucleic Acids. 2024 Jul 19;35(3):102282. doi: 10.1016/j.omtn.2024.102282 (PMC11339036; doi:10.1016/j.omtn.2024.102282)
Supplement: Document S1. Figures S1–S5 and Tables S1–S5 [file mmc1.pdf]

OMTN, Volume *35*

Supplemental information

Suppression of HSV-1 infection and viral reactivation by CRISPR-Cas9 gene editing in 2D and 3D culture models

Anna Bellizzi, Senem Çakır, Martina Donadoni, Rahsan Sariyer, Shuren Liao, Hong Liu, Guo-Xiang Ruan, Jennifer Gordon, Kamel Khalili, and Ilker K. Sariyer

Table S1. HSV-1 ICP0 and ICP27 specific gRNA sequences

gRNAs name	nucleotide positions (RefSeq NC_001806.2)	nucleotide positions (GenBank MF959544.1)	Target strand	Sequence (+ PAM sequence)
ICP27m1	115156-115181	115045-115064	plus	5'-AATCCTAGACACGCACCGCC**AGGAGT**-3'
ICP27m2	114376-114401	114271-114290	minus	5'-**TGGGGG**GGGCGATTACTGCGACCGCT-3'
ICP0m1	5319-5344	5180-5199	plus	5'-GTACCCGACGGCCCCCGCGT**CGGAGT**-3'
ICP0m2	4496-4521	4312-4331	plus	5'-CTCAGGCCGCGAACCAAGAA**CAGAGT**-3'
ICP0m1	121030-121055	121086-121105	minus	5'-**TGAGGC**TGCGCCCCCGGCAGCCCATG-3'
ICP0m2	121853-121878	121954-121973	minus	5'-**TGAGAC**AAGAACCAAGCGCCGGACTC-3'

Table S2. Excision Assay primers

Primer name	nucleotide positions (RefSeq NC_001806.2)	nucleotide positions (GenBank MF959544.1)	Primer sequence
ICP27_**PF**	113735-113756	113624-113645	5’-ATGGCGACTGACATTGATATGC-3’
ICP27_**PR**	115187-115206	115076-115095	5’-GTCAACTCGCAGACACGACT-3’
ICP0_**PF**	4231-4256/<br>122118-122143	4047-4072/<br>122213-122238	5’-AACAACAGAGACCCCATAGTGAT-3’
ICP0_**PR**	5470-5488/<br>120886-120904	5331-5349/<br>120936-120954	5’-ATTGTTTTCCCTCGTCCCG-3’

Table S3. HSV-1 ICP0 and ICP27 specific gRNA sequence for cloning in SaCas9/gRNA expression plasmid pX601-AAV-CMV::NLS-SaCas9-NLS-3xHA-bGHpA;U6::BsaI-sgRNA (px601)

Primer name	Sequence
gRNA_ICP27m1_Fw_clon	5'-CACCG AATCCTAGACACGCACCGCC-3'
gRNA_ICP27m1_Rev_clon	5'-AAAC GGCGGTGCGTGTCTAGGATT C-3'
gRNA_ICP27m2_Fw_clon	5'-CACCG TCGCCAGCGTCATTAGCGGG-3'
gRNA_ICP27m2_Rev_clon	5'-AAAC CCCGCTAATGACGCTGGCGA C-3'
gRNA_ICP0m1_Fw_clon	5'-CACCG GTACCCGACGGCCCCCGCGT-3'
gRNA_ICP0m1_Rev_clon	5'-AAAC ACGCGGGGGCCGTCGGGTAC C-3'
gRNA_ICP0m2_Fw_clon	5'-CACCG CTCAGGCCGCGAACCAAGAA-3'
gRNA_ICP0m2_Rev_clon	5'-AAAC TTCTTGGTTCGCGGCCTGAG C-3'
T795_Fw_In-Fusion_U6::gRNA	5'-ATT ACG CTT AAG AAT TCC TAG AGC-3'
T796_Rev_In-Fusion_U6::gRNA	5'-GGA AAT AGG CCC TCA GAC TAG GGG TTC CTG CGG CCG CAA A-3'

Table S4. HSV-1 ICP0 and ICP27 gRNA and SaCas9 RT-qPCR primers

**Primer name**	**Sequence**
RT_gRNA_px60_scaffold_Rev	5'-CGC CAA CAA GTT GAC GAG ATA A-3'
ICP0m1_gRNA_RT_Fw	5'-ATA TGT ACC CGA CGG CCC CCG C-3'
ICP0m2_gRNA_RT_Fw	5'-CTC AGG CCG CGA ACC AAG AA-3'
ICP27m1_gRNA_RT_Fw	5'-AAT CCT AGA CAC GCA CCG CC-3'
ICP27m2_gRNA_RT_Fw	5'-TCG CCA GCG TCA TTA GCG GG-3'
RT_gRNA_px60_scaffold_FAM_Probe	5'-CAG AAT CTA CTA AAA CAA GGC AAA-3'
SaCas9_RT_Fw	5'-CCG AGT TCA CCA ACC TGA A-3'
SaCas9_RT_Rev	5'-GTC AGC TCG GAG TTC AGA TTG-3'
SaCas9_RT_FAM_Probe	5'-AAC GCC GAG CTG CTG GAT CA-3'
Macaca mulatta beta-actin_RT_Fw	5'-TGG ACT TCG AGC AGG AGA TG-3'
Macaca mulatta beta-actin_RT_Rev	5'-GAA GGA AGG TTG GAA GAG AG-3'
Macaca mulatta beta-actin_RT_HEX_Probe	5'-CGG CGG CTT CCA GCT CCT CC-3'

Table S5. HSV-1 LAT, gBp and ICP27 RT-qPCR and ddPCR primers

Primer name	nucleotide positions (GenBank MF959544.1)	Sequence
LAT_Intron_cDNA_Rev	6588-6607/ 119678-119697	5'-GTG GTC GGA CGG GTA AGT AA-3'
LAT_intron_Fw	6692-6708/ 119577-119593	5'-CGC CCC AGA GGC TAA GG-3'
LAT_intron_Rev	6645-6663/ 119622-119640	5'-GGG CTG GTG TGC TGT AAC A-3'
HSV-1_ICP27_Fw	113757-113776	5'-CGT CGG ACG AGG ACA TGG AA-3'
HSV-1_ICP27_Rev	113905-113924	5'-GCG CTG GTT GAG GAT CGT TG-3'
HSV-1_gBp_Fw	53688-53707	5'-CGC TGG ACC TCC GTG TAG TC-3'
HSV-1_gBp_Rev	53793-53811	5'-CCG TCA GCA CCT TCA TCG A -3'
HSV-1_gBp_FAM_Probe	53713-53735	5'-6-FAM/CC ACG AGA T/ZEN/C AAG GAC AGC GGC C/3IABkFQ-3'
*Homo sapiens*_beta-actin_SYBR_Fw	none	5'-CCT CGC CTT TGC CGA TCC-3'
*Homo sapiens*_beta-actin_SYBR_Rev	none	5'-CGC GGC GAT ATC ATC ATC C-3'
*Homo sapiens*_TERT_Fw	none	5'-TGG AGC AAG TTG CAA AGC AT-3'
*Homo sapiens*_TERT_Rev	none	5'-CAG AGC CTT GCA CAG AAT CC-3'
*Homo sapiens*_TERT_HEX_Probe	none	5'-5-HEX/CCG GCC TCA/ZEN/GCA TGC GCC TG/3IABkFQ-3'

Figure S1

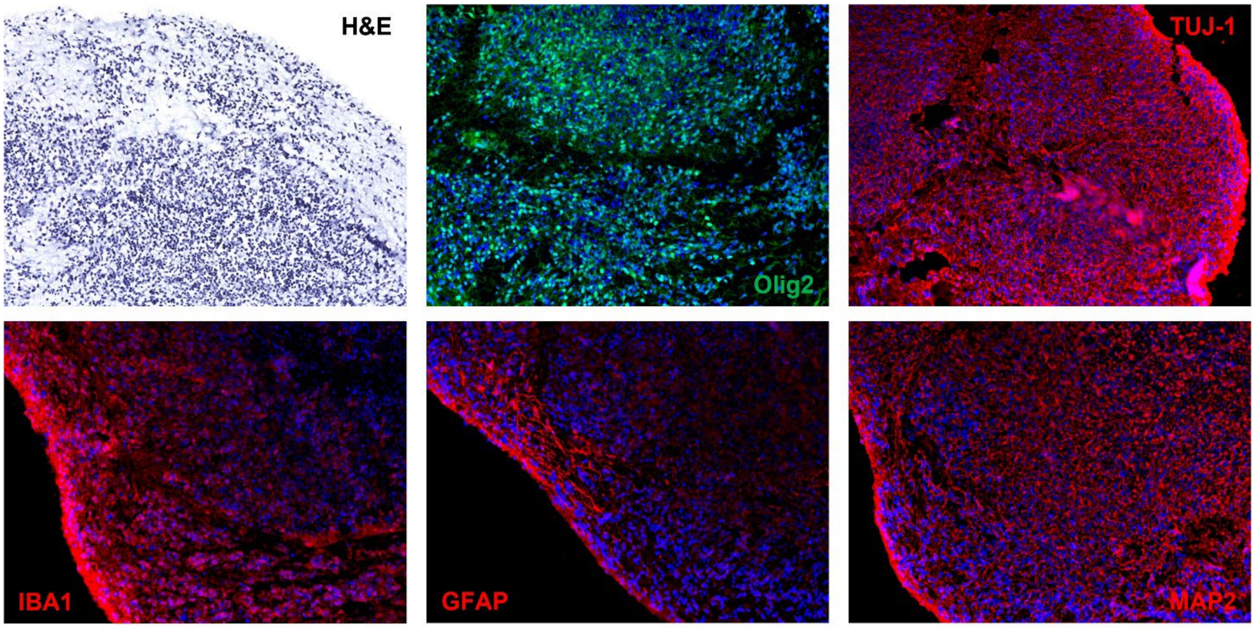

Cerebral organoids (COs) characterization. COs sections of 10μm were stained with hematoxylin and neurons, microglia, astrocytes, and oligodendrocytes were visualized by immunohistochemistry utilizing specific antibodies for MAP2/TUJ-1, IBA1, GFAP, and Olig2, respectively. Microtubule-associated protein 2 (MAP2) is a neuron-specific cytoskeletal protein, enriched in dendrites and with an essential role in determining and stabilizing neuronal morphology during neuron development. Class III beta-tubulin (TUJ-1) is expressed in premature neurons and is used as a marker to identify neural differentiation in the developing brain. Ionized calcium-binding adaptor molecule 1 (IBA1) is a calcium-binding protein and is specifically expressed in microglia in the brain. Glial fibrillary acidic protein (GFAP) is an intermediate filament-III protein uniquely found in astrocytes in the central nervous system. The oligodendrocyte transcription factor 2 (Olig2) activates the expression of myelin-associated genes in the oligodendrocyte-lineage cells and thus it represents a valid marker for oligodendrocytes.

Figure S2

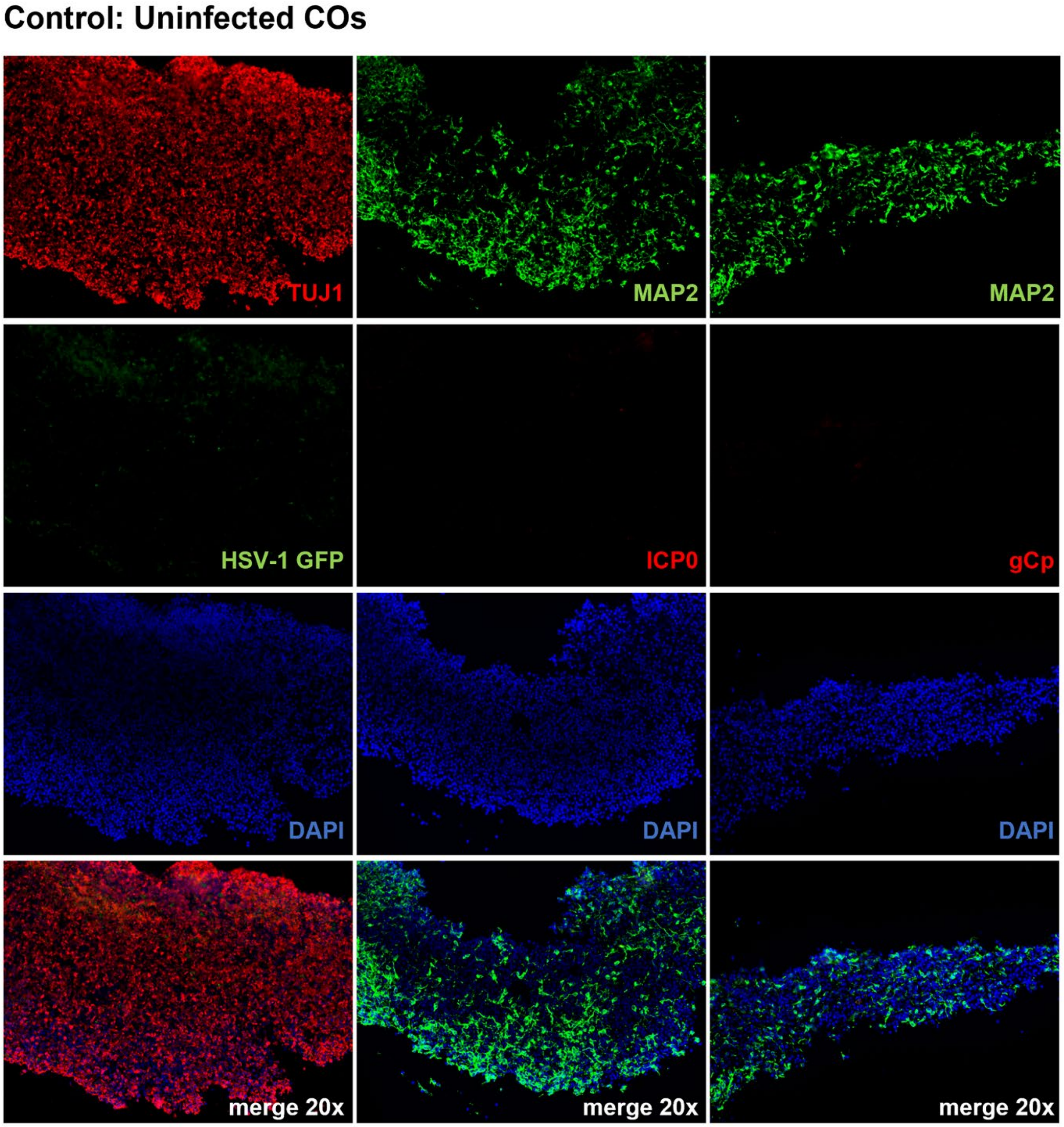

Immunohistochemistry of uninfected COs, as control**s** of HSV-1 infects COs (Figure 3E and 3F) and HSV-1 Latency Model in COs (Figure 4C and 4D). These organoids resulted positive to the staining with the neuronal markers MAP2 and TUJ-1, and negative for the staining with specific antibodies for the GFP protein expressed by the HSV-1 Patton strain and the HSV-1 proteins ICP0 and gCp.

Figure S3

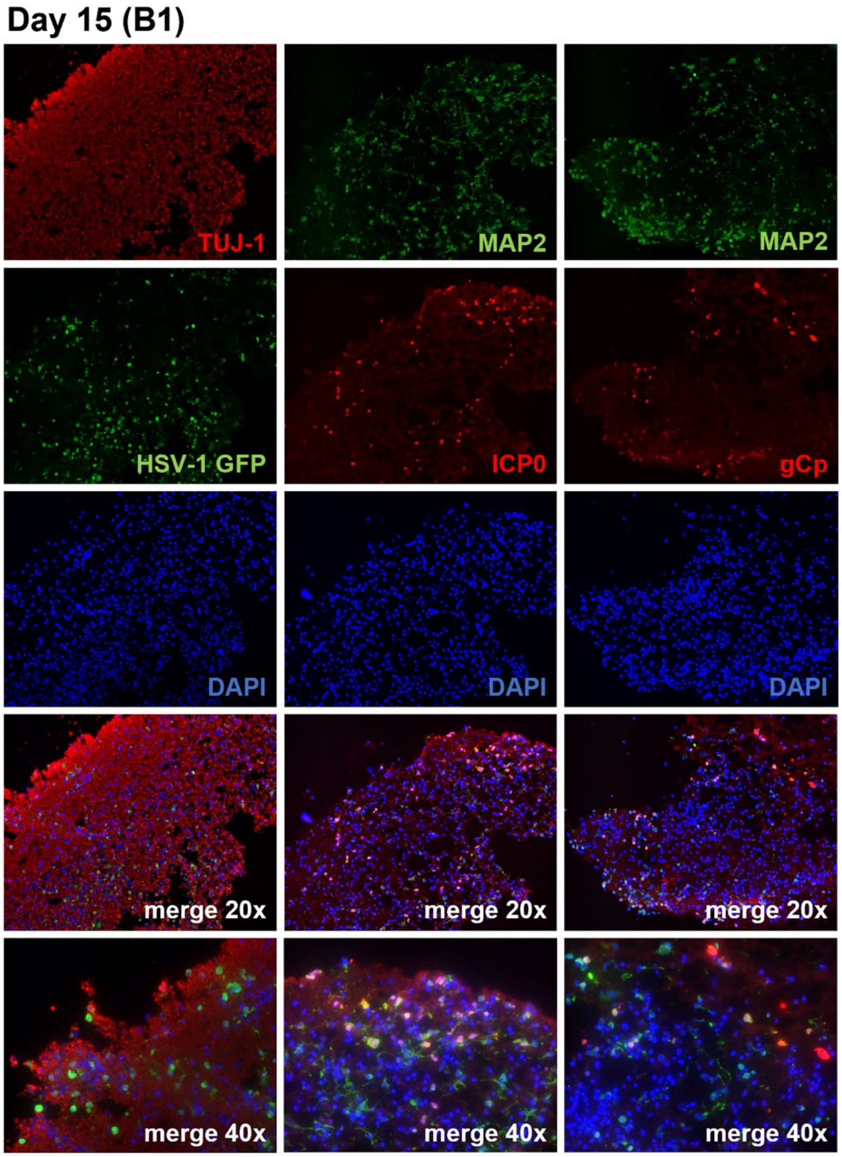

Immunohistochemistry of COs at 15 days post-infections (at day 4 of induction of HSV-1 GFP reactivation by LY294002 and 11 days of latency establishment by Valacyclovir). Viral protein gCp, ICP0 and HSV-1 GFP were stained in combination with the neuronal markers MAP2 and TUJ-1 at day 15 of the HSV-1 Latency Model timeline (Figure 4).

Figure S4

A.

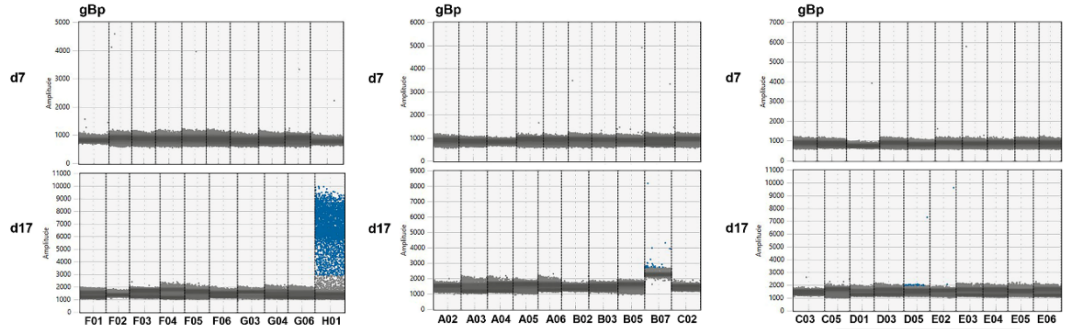

Organoids	Timepoint HSV+ untreated	
	d7	d17
	Copies/mL (ddPCR)	Copies/mL (ddPCR)
F01	0	0
F02	0	0
F03	0	0
F04	0	0
F05	0	0
F06	0	0
G03	0	0
G04	0	0
G06	0	0
H01	0	4.20E+6

Organoids	Timepoint HSV+ AAV2-ICP0gRNA	
	d7	d17
	Copies/mL (ddPCR)	Copies/mL (ddPCR)
A02	0	0
A03	0	0
A04	0	0
A05	0	0
A06	0	0
B02	0	0
B03	0	0
B05	0	0
B07	0	5.20E+4
C02	0	0

Organoids	Timepoint HSV+ AAV2-ICP27gRNA	
	d7	d17
	Copies/mL (ddPCR)	Copies/mL (ddPCR)
C03	0	0
C05	0	0
D01	0	0
D03	0	0
D05	0	4.60E+4
E02	0	2.80E+3
E03	0	0
E04	0	0
E05	0	0
E06	0	0

B.

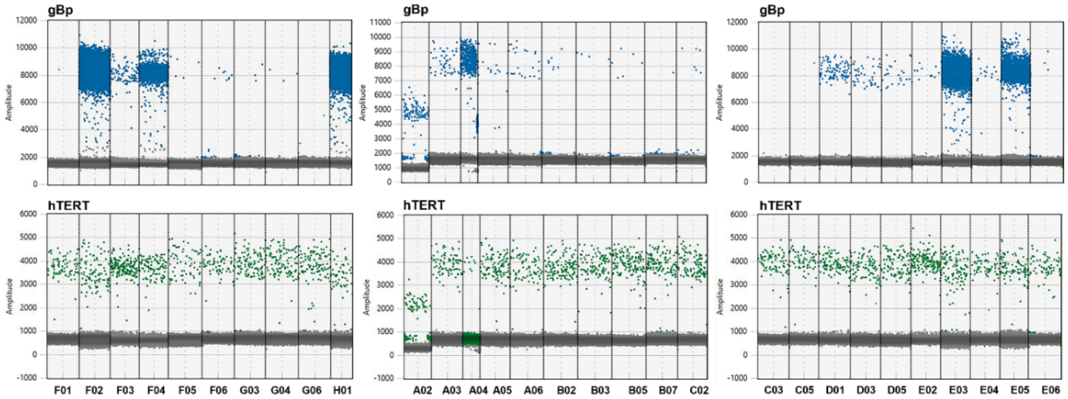

Organoids	HSV+ infection untreated
F01	0
F02	1.24E+6
F03	1.08E+4
F04	6.81E+5
F05	1.95E+3
F06	5.91E+3
G03	4.23E+3
G04	7.06E+2
G06	0
H01	2.57E+6

Organoids	HSV+ AAV2-ICP0gRNA
A02	2.26E+4
A03	1.19E+4
A04	5.89E+2
A05	3.38E+3
A06	6.67E+3
B02	2.28E+3
B03	1.51E+3
B05	2.82E+3
B07	1.74E+3
C02	2.24E+3

Organoids	HSV+ AAV2-ICP27gRNA
C03	0
C05	0
D01	1.76E+4
D03	9.83E+3
D05	4.64E+3
E02	1.56E+3
E03	5.69E+5
E04	5.45E+3
E05	4.26E+5
E06	3.88E+3

Quantification of HSV-1 GFP DNA by Digital Droplet PCR (ddPCR) from Figure 5 (**A**) Digital droplet PCR (ddPCR) traces of HSV-1 genome copies number in COs culture media at the timepoints day 7 (d7) and day 17 (d17). (**B**) ddPCR traces of HSV-1 gBp and cellular TERT internal control at day 21 to calculate the HSV-1 genome copies number per 20,000 cells.

Figure S5

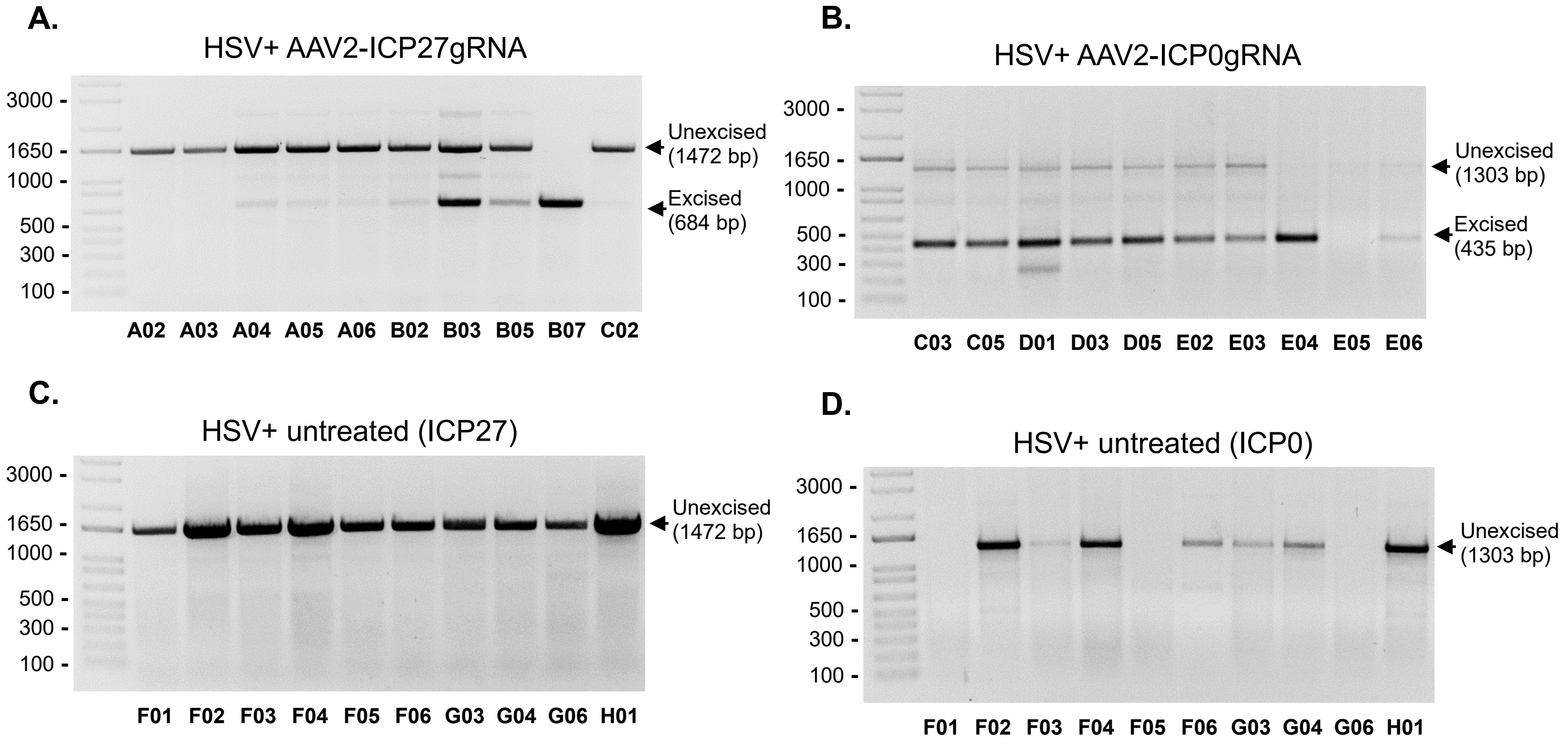

Excision Assay for ICP27 and ICP0 in COs treated with AAV2-CRISPR/Cas9 editing platforms. Excision assay was performed on HSV-1 ICP27 (**A**) and ICP0 (**B**) sequences from COs infected with HSV-1 and transduced with AAV2_CRISPR constructs. Same PCR reactions were also performed for HSV-1 ICP27 (**C**) and ICP0 (**D**) in COs infected with HSV-1 only.
